# Transdiagnostic metacognitive therapy compared to disorder-specific cognitive behavioral therapy for anxiety disorders: a study protocol for a randomized controlled superiority trial

**DOI:** 10.1186/s13063-026-10063-4

**Published:** 2026-10-30

**Authors:** Nathalie Petersén, Fredrik Santoft, Nitya Jayaram-Lindström, Markus Jansson-Fröjmark, Sven Alfonsson, Benjamin Bohman

**Affiliations:** Centre for Psychiatry Research, Department of Clinical Neuroscience, Karolinska Institutet, & Stockholm Health Care Services, Region Stockholm, Stockholm, Sweden

**Keywords:** Metacognitive therapy, Cognitive behavioral therapy, Transdiagnostic treatment, Anxiety disorders

## Abstract

**Background:**

Anxiety disorders are highly prevalent worldwide and are associated with functional impairment, reduced quality of life and high societal costs. With only approximately 50% of patients achieving remission following cognitive behavioral therapy (CBT), the evaluation of other effective treatments is warranted. Studies suggest that metacognitive therapy (MCT) may be more effective than CBT for anxiety disorders. Although transdiagnostic treatment may be beneficial for treatment implementation and dissemination in routine care, there is currently only one adequately powered study comparing transdiagnostic MCT (tMCT) to disorder-specific CBT for anxiety disorders. The main aim of this trial is to investigate the effectiveness of tMCT compared to disorder-specific CBT for anxiety disorders. The trial also aims to investigate potential mediators of treatment effects in tMCT and CBT, to conduct a health economic evaluation and to explore patients' perspectives on the treatments.

**Methods:**

This trial is a prospective, pragmatic, two-arm parallel-group randomized controlled superiority trial comparing tMCT with disorder-specific CBT, conducted in outpatient psychiatric services in Stockholm, Sweden. A sample of 86 adults with a principal diagnosis of generalized anxiety disorder, social anxiety disorder or posttraumatic stress disorder will be recruited and randomly allocated to tMCT or CBT. Primary outcomes are self-rated symptoms of principal diagnosis, and secondary outcomes are clinical severity, remission rates, depressive symptoms, functional impairment and quality of life. Outcomes will be assessed at pre- and post-treatment as well as at 6- and 12-month follow-up. Metacognitions and cognitions as potential mediators will be measured weekly during treatment. Cost-effectiveness of tMCT compared to CBT will be investigated. Semi-structured interviews will be conducted with participants from both conditions.

**Discussion:**

Considering the potential of MCT, further research is needed to evaluate its effectiveness compared to CBT, particularly MCT in a transdiagnostic format. Results from this trial will add to the evidence base for tMCT and inform treatment development, decision-making and implementation of effective treatment for anxiety disorders in routine care.

**Trial registration:**

ClinicalTrials.gov NCT06937892. Registered on April 22nd, 2025

## Introduction

### Background and rationale {6a}

Anxiety disorders are highly prevalent around the world, with an estimated global lifetime prevalence of 11.3% in males and 18.8% in females [1]. These disorders include generalized anxiety disorder (GAD), social anxiety disorder (SAD), and anxiety-related disorders such as posttraumatic stress disorder (PTSD; further referred to as an anxiety disorder for simplicity). In Sweden, every fourth individual develops an anxiety disorder during their lifespan, with a 2–3 times higher risk in females than males [2]. In addition to psychological distress, anxiety disorders are associated with impairment in social, occupational and physical functioning [3], reduced quality of life [4] and large societal costs [5, 6].

Cognitive behavioral therapy (CBT) is an effective treatment for anxiety disorders [7], recommended as the first-line treatment in clinical guidelines [2, 8]. Despite this, only approximately 50% of patients with anxiety disorders achieve remission status following CBT [9], stressing the need for research on more effective treatment options. The current evidence for CBT relies mainly on research on disorder-specific treatment protocols [7]. However, research on transdiagnostic treatment approaches has emerged, in which one single protocol is used to treat multiple disorders [10–12]. Transdiagnostic treatment has the potential to improve the implementation and dissemination of psychological treatment in routine care. For example, training in a single transdiagnostic protocol, rather than multiple disorder-specific protocols, may facilitate adequate therapist skill development in clinical routine settings [10]. Transdiagnostic treatment may also improve treatment adaptation in populations with high comorbidity while maintaining treatment fidelity [13].

Metacognitive therapy (MCT) is a relatively new psychological treatment with theoretical foundations that are compatible with a transdiagnostic framework [14]. MCT is based on the self-regulatory executive function (S-REF) model [12], a generic theoretical framework emphasizing the role of dysfunctional negative and positive metacognitions in the development and maintenance of anxiety and other psychological complaints. Metacognitions refer to cognitions about cognitions (e.g., “I cannot control my thoughts” or “Focusing on danger will keep me safe”), distinct from cognitions that are commonly targeted in CBT (e.g., “What if something bad happens?” or “I do not feel safe”). According to the S-REF model, dysfunctional metacognitions influence responses to triggers (automatic thoughts) through activating the cognitive attentional syndrome (CAS). The CAS involves responding to triggers with strategies such as worry, rumination, threat monitoring and maladaptive coping behaviors. Use of the CAS affects emotional responses and contributes to new triggers, thus prolonging and maintaining psychological distress. In MCT, the primary focus is on reducing or eliminating the CAS by targeting the controlling metacognitions [15]. Several studies have provided support for the S-REF model. For example, dysfunctional metacognitions have shown to be associated with symptoms of anxiety and depression [14], are elevated in psychiatric patients as compared to healthy individuals [16] and have been found to predict subsequent anxiety symptoms [17]. Furthermore, large reductions in dysfunctional metacognitions have been observed following MCT across studies [18] and following CBT, although to a lesser extent [19]. In addition, there are a few studies that provide preliminary support for metacognitions as a mediator of treatment response in both MCT and CBT [20, 21]. This suggests the clinical relevance of targeting metacognitions in the treatment of anxiety disorders; however, more studies are needed.

Meta-analyses have indicated that MCT may be superior to CBT for various psychiatric disorders [18, 22]. The most recent meta-analysis conducted in 2024 included 14 studies comparing MCT and CBT [22]. In favor of MCT, a between-group effect size of Hedge’s *g* = 0.43 was observed post-treatment across disorders [22]. Still, more studies with larger samples conducted in different settings are warranted to draw firm conclusions about the effectiveness of MCT. In addition, although the S-REF model provides a generic framework for a transdiagnostic treatment approach [14], MCT has mainly been investigated in a disorder-specific format. There is currently only one adequately powered study comparing transdiagnostic MCT (tMCT) to disorder-specific CBT for anxiety disorders [23]. The study included participants with panic disorder, SAD and PTSD in an inpatient setting. A between-group effect size of Cohen’s *d* = 0.70 on anxiety symptoms was found at post-treatment, favoring tMCT. Considering the limited number of studies, it is highly warranted to further investigate the effectiveness of tMCT compared to CBT in different settings.

MCT might also have health economic benefits compared to CBT. Recently, the first health economic evaluation of MCT was published, suggesting that the treatment is cost-effective [24]. Some studies indicate that MCT requires fewer sessions [25] and shorter average session time [23] than CBT, while being the more effective treatment. Thus, MCT might be a more cost-effective use of resources compared to CBT. To date, no health economic evaluation of MCT has been conducted in a psychiatric setting and the cost-effectiveness of MCT compared to CBT is yet to be investigated.

To inform future treatment development and implementation, qualitative studies on patient perceptions of MCT are warranted. At present, qualitative studies on MCT remain scarce and have been conducted mainly in non-psychiatric populations. MCT has been reported as an intervention that is well understood and positively received [26]. Also, case conceptualization in MCT was found to be more parsimonious and easier to apply than case conceptualization in CBT in a sample of cardiac patients with emotional distress, due to the simplicity of addressing repetitive negative thinking as a single construct rather than distinguishing between realistic and unrealistic thoughts [27]. Nevertheless, patient perspectives on MCT, and how it is experienced relative to CBT, remain insufficiently examined in routine psychiatric care. This underscores the need for further qualitative and comparative research in psychiatric services.

### Objectives {7}

The main aim of this trial is to evaluate the short- and long-term effects of tMCT compared to disorder-specific CBT for anxiety disorders. Secondly, the trial aims to examine metacognitions and cognitions as possible mediators of change to contribute to the knowledge base on what makes psychological treatment work. A third aim is to investigate the cost-effectiveness of tMCT compared to CBT. Lastly, the trial aims to explore participants’ experiences of tMCT and CBT to help inform treatment development, adaptation and implementation in routine care.

Based on the recent meta-analysis [22] and the previous study on tMCT [23], the main hypothesis is that tMCT will be superior to CBT at post-treatment on the primary outcome measure of symptoms of principal diagnosis and on secondary outcome measures of clinical severity, remission rates, depressive symptoms, functional impairment and quality of life. It is also expected that tMCT will remain superior at 6- and 12-month follow-up assessments. Further, it is expected that change in metacognition is a stronger mediator of treatment response than change in cognition. Finally, tMCT is hypothesized to be more cost-effective relative to CBT. As the qualitative study has an exploratory aim, no hypothesis was formulated.

### Trial design {8}

This is a prospective, pragmatic, two-arm parallel-group randomized controlled superiority trial, designed to compare tMCT with disorder-specific CBT. Participants will be randomly assigned with a 1:1 allocation ratio to either tMCT or CBT. Additionally, the trial includes 6- and 12-month follow-up assessments after end of treatment, an investigation of potential mediators, a cost-effectiveness analysis and a qualitative study.

## Methods: participants, interventions, and outcomes

### Study setting {9}

The trial will be conducted in psychiatric outpatient care centers at a publicly funded psychiatric clinic in Stockholm, Sweden. A list and description of study sites can be obtained from the corresponding author.

### Eligibility criteria {10}

The trial population of interest is adult patients in psychiatric outpatient routine services. For enrollment, participants will be required to fulfill the following inclusion criteria: (1) 18 years of age or older, (2) a principal (most interfering and/or severe) diagnosis of GAD, SAD, or PTSD, assessed using the semi-structured Diagnostic Interview for Anxiety, Mood and OCD and Related Neuropsychiatric Disorders [28], (3) if on psychotropic medication, no change in dose during the last 6 weeks, and (4) ability to read and speak Swedish. Participants are required not to fulfill any of the following exclusion criteria: (1) a current diagnosis of psychotic disorder, bipolar disorder, neurocognitive disorder or moderate to severe substance use disorder, (2) elevated or acute risk of suicide (such as suicidal plans or intent that would require acute and adapted intervention for suicide prevention), and (3) simultaneous psychological treatment. There are no other restrictions on comorbidity.

A principal diagnosis of GAD, SAD, or PTSD is chosen for eligibility to reflect the service population of psychiatric outpatient care in Stockholm, Sweden. Other anxiety disorders are excluded as these are either more commonly treated at the primary care level (such as panic disorder and specific phobia) or treated in disorder-specific psychiatric outpatient care centers (such as obsessive-compulsive disorder).

For the qualitative study, participants who received treatment in the trial will be invited to take part in semi-structured individual interviews about their experiences of the respective treatment.

#### Therapist eligibility and training

Trial therapists are licensed psychologists with basic training in CBT, employed within routine psychiatric outpatient services. Experience in training and practice of psychological treatment will be reported for all therapists in future publications. Therapists will provide either tMCT or CBT. Before enrollment, therapists will be required to demonstrate adequate competence in their allocated treatment.

Therapists providing tMCT will attend a 3-day didactic and skill-building workshop on MCT; 2 days on standard MCT and 1 day on the transdiagnostic protocol. The MCT workshop will be led by licensed psychologists with level 2 MCT training and extensive prior experience in MCT practice, training and supervision. Before the workshop, therapists will be required to read literature on MCT [15, 29] to acquire sufficient theoretical understanding. Following the workshop, therapists will provide a complete treatment using the tMCT protocol with a training patient with a principal diagnosis of GAD. All training sessions will be audio-recorded, and therapists will receive weekly individual supervision for up to 14 sessions. Supervision sessions will be based on weekly competence ratings of therapist performance, with a focus on increasing MCT competence and protocol adherence. For tMCT therapist enrollment, supervisors will assess therapist competence based on audio recordings of three pre-specified sessions with the training patient.

As all trial therapists have prior basic CBT training, CBT therapists will attend a five-hour workshop on adherence to the disorder-specific CBT protocols. For enrollment of CBT therapists, independent assessors will assess competence based on audio recordings of one pre-specified session with three patients with a principal diagnosis of GAD, SAD, or PTSD.

#### Competence assessors and supervisors

Competence assessors and supervisors in the trial are licensed psychologists and psychotherapists specialized in CBT, with advanced training (level 2) in MCT. The assessors and supervisors have extensive experience in clinical practice, training and supervision of MCT and/or CBT, the extent of which will be reported in future publications.

### Who will take informed consent? {26a}

Therapists will provide eligible patients with oral and written information about the trial. Patients will be given adequate time to review the written materials and to ask questions. If needed, patients will be given additional time to decide whether they would like to participate. Individuals who choose to participate will provide written informed consent, obtained by therapists.

### Additional consent provisions for collection and use of participant data and biological specimens {26b}

Therapists will collect additional written informed consent from participants who wish to partake in the qualitative study.

## Interventions

### Explanation for the choice of comparators {6b}

Disorder-specific CBT is chosen as the comparator due to its well-established efficacy for anxiety disorders [7] and because it is recommended as the first-line treatment in clinical guidelines [2, 8]. The chosen disorder-specific CBT protocols for GAD, SAD and PTSD are empirically supported [30–35] and commonly seen as gold standard treatments in routine care.

### Intervention description {11a}

Participants in both conditions will partake in weekly, individual face-to-face treatment sessions. Each session includes reviewing the previous week’s homework assignment, implementing verbal- and/or experience-based in-session interventions and developing new homework assignments.

Both tMCT and CBT will be provided for a minimum of 8 and a maximum of 13 sessions, with the exact number determined by the therapist’s clinical judgement of optimal treatment effect. This range is chosen to enable controlling for treatment dose while promoting equitable conditions for both treatments, balancing the typically shorter MCT (e.g., 5–10 sessions recommended for GAD) [15]) against the longer CBT protocols (8–15 sessions recommended for PTSD [36]; 12–14 sessions commonly used for GAD [19, 31]; up to 14 sessions recommended for SAD [37].) The standard session duration is set to 45 min, with the option to extend up to 90 min if needed for in-session interventions, such as exposure exercises or behavioural experiments. In prolonged exposure for PTSD, up to 90 min is allowed for all sessions in accordance with the treatment manual [36]. The mean number of sessions and mean session duration will be reported for both tMCT and CBT in future publications.

#### Transdiagnostic metacognitive therapy

For this trial, a transdiagnostic protocol was developed based on the general treatment principles and specifications for transdiagnostic treatment as described by Wells [15]. In addition, a transdiagnostic case conceptualization was created in accordance with the universal formulation as outlined by Wells [15]. The tMCT protocol consists of 8 modules focused on reducing the CAS, modifying dysfunctional negative and positive metacognitions that maintain the CAS and implementing new plans for processing thoughts. The protocol is structured and includes the following key components:

1. Case conceptualization and socialization to the treatment model
2. Detached mindfulness and worry/rumination postponement
3. Modifying negative metacognitive beliefs about the uncontrollability of worry and/or rumination
4. Removing threat monitoring through Situational Attentional Refocusing and/or Spatial Attentional Control Exercise
5. Removing maladaptive coping behaviors (e.g., avoidance, thought suppression, reassurance seeking, substance use)
6. Modifying negative metacognitive beliefs about the danger of worry and/or rumination
7. Modifying positive metacognitive beliefs
8. Relapse prevention: Summary of old plan and implementation of new plan

#### Disorder-specific cognitive behavioral therapy

In the CBT condition, participants will receive CBT according to disorder-specific protocols. Intolerance-of-uncertainty therapy [38] is used for GAD, cognitive therapy [39] for SAD, and prolonged exposure [36] for PTSD.

### Criteria for discontinuing or modifying allocated interventions {11b}

Treatment will be discontinued upon any participant’s request or in the event of withdrawal of consent. Treatment will be paused or discontinued in cases of acute conditions or deterioration that requires other immediate interventions.

### Strategies to improve adherence to interventions {11c}

Participant adherence will be continuously monitored by their therapist. This enables problem-solving in cases of low treatment adherence, such as with homework assignments. During the treatment phase, therapists will receive supervision in tMCT or CBT twice a month to ensure maintained competence and protocol adherence.

### Relevant concomitant care permitted or prohibited during the trial {11d}

During the trial period, participants will be requested not to engage in other psychological treatment. All other concomitant care is permitted. Participants will be encouraged not to make any changes to any psychotropic medication.

### Provisions for post-trial care {30}

There will be no provisions for post-trial care. Ancillary or post-trial care will be provided if needed at the psychiatric clinic according to routine clinical guidelines.

### Outcomes {12}

#### Primary outcome

- Change in core symptoms of principal diagnosis, assessed using self-rated disorder-specific measures. To test the main hypothesis that tMCT is superior to CBT, the outcome will be evaluated at baseline, post-treatment, and at 6- and 12-month follow-up.

#### Secondary outcomes

- Change in clinician-rated clinical severity and remission status of principal and comorbid diagnosis, from baseline to post-treatment. Remission at post-treatment is defined as no longer meeting diagnostic criteria.
- Change in self-rated depressive symptoms, functional impairment and quality of life, evaluated at baseline, post-treatment, and at 6- and 12-month follow-up

#### Process outcomes

- Potential mediation of treatment effect on general anxiety by changes in metacognition and/or cognition, assessed using self-rated measures weekly during treatment.

#### Health economic outcomes

- Cost-effectiveness assessed using self-rated medical costs and productivity losses, quality-adjusted life years and the primary outcome at baseline, post-treatment, 6- and 12-month follow-up.

#### Qualitative outcome

- Participant perceptions of tMCT and CBT as treatment models, explored using semi-structured interviews conducted up to 1 month after treatment completion.

### Plans for assessment and collection of outcomes {18a}

#### Primary outcome measures

For the primary outcome, the chosen measures are the gold standard self-report measures for symptom ratings of the respective disorders. A blinded clinician-administered disorder-specific symptom rating scale for each diagnosis, such as the Clinician-Administered PTSD Scale for DSM-5 [40], would constitute a more optimal primary outcome measure. However, to our knowledge, no equivalent measure currently exists for GAD or SAD. The choice of self-rated measures for the primary outcome is also consistent with prior MCT trials [22], facilitating comparison.

For participants with a principal diagnosis of GAD*,* the Penn State Worry Questionnaire (PSWQ) [41] will be used. The PSWQ is a 16-item self-report measure of symptoms of worry, assessing their frequency, intensity and uncontrollability. Participants rate how characteristic each item is for them on a 1–5 scale (1 = Not at all typical for me, 5 = Very typical for me). The scale has demonstrated high internal consistency (Cronbach’s α = 0.91–0.95) as well as good test-retest reliability (*r* = 0.83) [41]. For participants with a principal diagnosis of SAD, the Liebowitz Social Anxiety Scale-Self-Report (LSAS-SR) [42] will be administered. The LSAS-SR items consist of 24 situations likely to elicit social anxiety. Each situation is rated on a 0–3 scale for levels of fear and avoidance during the past week (0 = None/never, 3 = Severe/usually). The scale has demonstrated high internal consistency (*α* = 0.95) [42] and good test-retest reliability (*r* = 0.83) [43]. For participants with a principal diagnosis of PTSD, the Posttraumatic Stress Disorder Checklist-5 (PCL-5) [44] will be used. The PCL-5 is a 20-item measure developed to mirror the DSM-5 criteria for PTSD directly. Respondents rate how much they have been bothered by each symptom during the past month on a 0–4 scale (0 = Not at all, 4 = Extremely). The PCL-5 has shown high internal consistency (*α* = 0.96) and good test-retest reliability (*r* = 0.84) [45].

#### Secondary outcome measures

Secondary outcomes are clinical severity of principal and comorbid diagnoses, remission rates, depressive symptoms, functional impairment, and quality of life. For assessment of principal and comorbid diagnoses, the Diagnostic Interview for Anxiety, Mood and OCD and Related Neuropsychiatric Disorders (DIAMOND) [28] will be administered before trial enrollment and at post-treatment. The DIAMOND is a clinician-administered semi-structured interview developed for diagnostic assessment of psychiatric disorders according to the Diagnostic and Statistical Manual of Mental Disorders, 5th edition (DSM-5) [46]. Clinical severity will be assessed using the clinician-administered 1-item Clinical Severity Rating (CSR) [47]*.* Clinical severity is rated for each diagnosis on a 0–8 scale (0 = No symptoms, 8 = Extremely severe symptoms). The scale has shown good inter-rater reliability for various anxiety disorders (*r* = 0.72–0.83) [48].

Symptoms of depression will be assessed using the 9-item Patient Health Questionnaire-9 (PHQ-9) [49]. Respondents rate how often they have experienced each symptom during the past two weeks on a 0–3 scale (0 = Not at all, 3 = Nearly every day). The PHQ-9 has demonstrated high internal consistency (*α* = 0.86–0.89) [49]. The World Health Organization Disability Assessment Schedule 2.0 (WHODAS 2.0) [50] will be used to assess functional impairment. In the 12-item self-report version, functional impairment over the last 30 days is measured through ratings of items covering six domains of functioning on a 0–4 scale (0 = No difficulty, 4 = Extreme or cannot do). The scale has demonstrated good psychometric properties when administered in populations with anxiety disorders, showing high internal consistency (*α* = 0.830.92) and test-retest reliability (Intraclass Correlation Coefficient = 0.83) [51]. To assess quality of life, the 5-item Satisfaction With Life Scale (SWLS) [52] will be administered. Items are rated on a 1–7 scale (1 = Strongly disagree, 7 = Strongly agree). When studied in a Swedish sample, the SWLS was shown to be a reliable measure with high internal consistency (*α* = 0.86) [53]. The 7-item Generalized Anxiety Disorder Scale (GAD-7) [54] is commonly used to assess the presence and severity of general anxiety. Respondents rate how often in the past 2 weeks they have experienced symptoms of anxiety on a 0–3 scale (0 = Not at all, 3 = Nearly every day). The GAD-7 has shown excellent internal consistency (*α* = 0.92) and good test-retest reliability (Intraclass Correlation Coefficient = 0.83) [54]. The GAD-7 will be administered weekly and used in mediation analysis.

#### Process measures

For evaluation of metacognitions and cognitions as potential mediators of treatment response, subscales of the Metacognitions Questionnaire-30 (MCQ-30) [55] and the Cognition Checklist [56] will be administered weekly during treatment. MCQ-30 is a measure of metacognition across five subscales. Participants rate their general agreement with the items on a 1–4 scale (1 = Do not agree, 4 = Agree very much). A five-factor solution has been obtained for the MCQ-30, one of which is the 6-item Uncontrollability and Danger subscale that targets negative metacognitive beliefs about the uncontrollability and danger of thoughts [57]. The subscale has demonstrated good convergent validity with pathological worry and anxiety [58], as well as high internal consistency (*α* = 0.83) [59]. The Cognition Checklist-Anxiety subscale (CCL-A) is a 12-item subscale of the Cognition Checklist that measures anxiety-related cognitions. A two-factor solution with depressive and anxiety-related cognitions has been obtained for the Cognition Checklist, supporting its subscales [56]. On the CCL-A, participants rate the occurrence of anxiety-related cognitions on a 1–5 scale (1 = Never, 5 = Always). CCL-A has shown good internal consistency (*α* = 0.89) [60].

#### Health economic measures

For evaluation of cost-effectiveness, costs will be assessed using the Treatment Inventory of Costs in Patients with psychiatric disorders (TIC-P) [61]. TIC-P measures self-rated medical costs and productivity losses during the past month. The scale is a feasible instrument for use in mental health populations and has demonstrated good test-retest reliability [62]. Effectiveness will be assessed using disorder-specific symptom ratings as the outcome measure. Also, cost-utility will be investigated; a type of cost-effectiveness analysis that uses quality-adjusted life years as the outcome. Quality-adjusted life years will be estimated from utility scores obtained by using the five-level EuroQol Five Dimensions (EQ-5D-5L) [63]. The EQ-5D-5L measures self-rated health across five domains. The utility score ranges from 0 (dead) to 1 (perfect health). The EQ-5D-5L has received extensive support as a valid and reliable instrument [64] and has been found responsive to change in anxiety symptoms [65].

#### Qualitative measure

A semi-structured interview guide with open-ended questions will be developed to gain insights into participants’ experiences of tMCT and CBT. The interview will cover topics such as the overall experience of the treatment, treatment usability and understanding of the treatment model. Examples of interview questions are “How did you experience the treatment?”, “Which parts of the treatment, if any, did you experience as positive or helpful?” and “Was there anything about the treatment that could have been different or better to be more helpful for you – and if so, what?” Initial responses will be explored further with probing follow-up questions.

#### Demographic and clinical data measures

A survey will be administered at baseline to collect demographic and anamnestic information (e.g., marital status and duration of symptomatology of principal diagnosis).

#### Therapist competence and adherence

Competence in MCT will be assessed using the 18-item Metacognitive Therapy Competency Scale (MCT-CS) [66]. Although the MCT-CS has not yet undergone psychometric evaluation, it is a standard measure in MCT research. Competence is rated on a 1–5 scale (1 = Very poor performance, 5 = Very good level). An item mean score of 3.0 on the MCT-CS (referred to in MCT-CS as a “mediocre” level) is chosen to reflect a minimum competence threshold for therapist enrollment. Competence in CBT will be assessed through the widely used 12-item Cognitive Therapy Scale-Revised (CTS-R) [67] on a 0–6 scale (0 = Absence of feature or highly inappropriate performance, 6 = Excellent performance or very good). An item mean score of 3.0 on the CTS-R is commonly considered a minimum competence standard [67] and will be used for enrollment of CBT therapists.

All treatment sessions will be audiotaped, and 20% will be randomly selected for assessment of therapist adherence to the treatment protocols. A random sample of sessions will be selected to assess inter-rater reliability.

### Participant timeline {13}

**Fig. 1:**
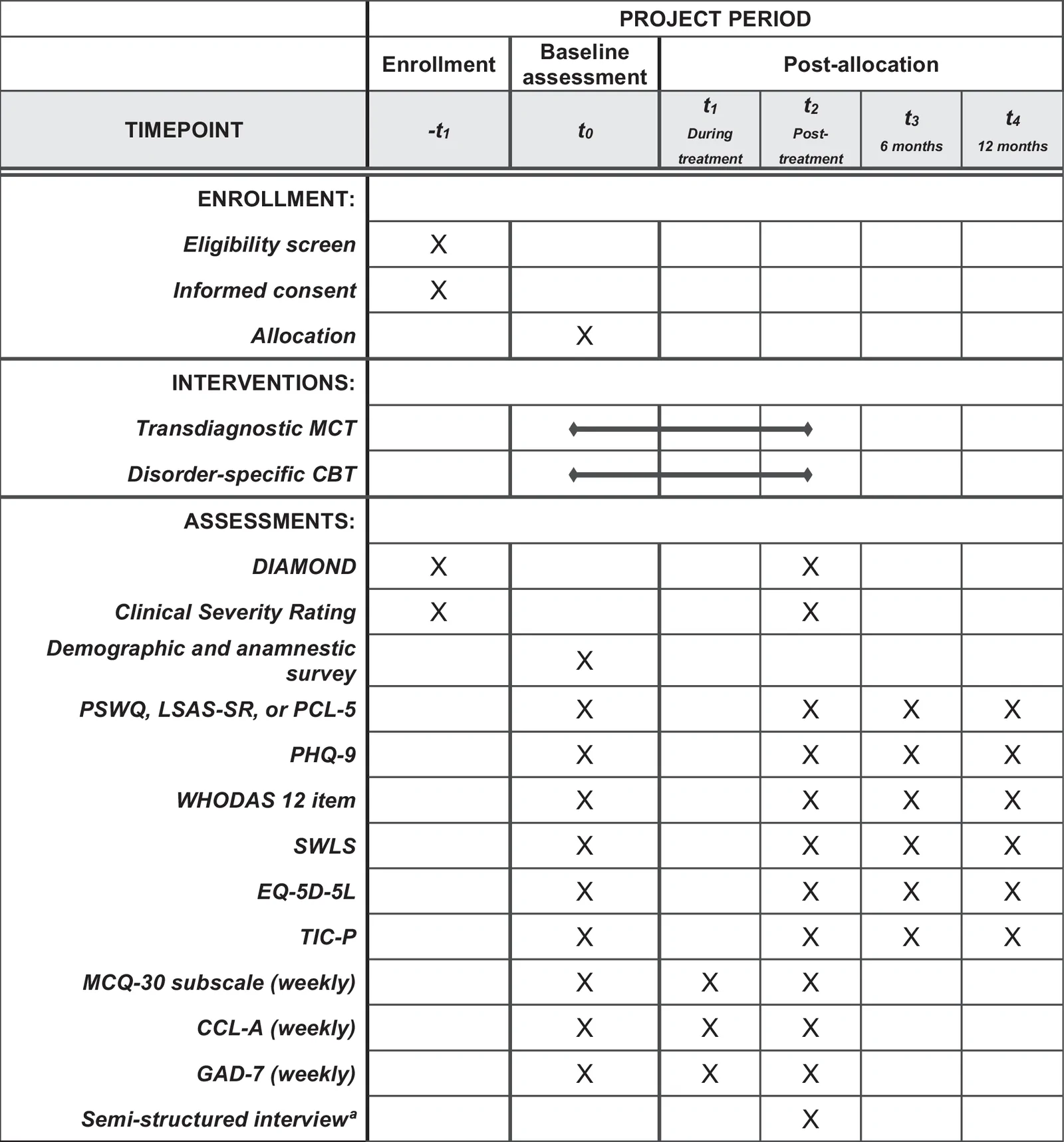
Schedule of enrollment, interventions, and assessments. Abbreviations: MCT, metacognitive therapy; CBT, cognitive behavioral therapy; DIAMOND, Diagnostic Interview for Anxiety, Mood, and OCD and Related Neuropsychiatric Disorders; PSWQ, Penn State Worry Questionnaire; LSAS-SR, Liebowitz Social Anxiety Scale-Self-Report; PCL-5, Posttraumatic stress disorder Checklist-5; PHQ-9, Patient Health Questionnaire-9; WHODAS, World Health Organization Disability Assessment Schedule; SWLS, Satisfaction With Life Scale; EQ-5D-5L, EuroQol Five Dimensions Five Levels; TIC-P, Treatment Inventory of Costs in Patients with psychiatric disorders; MCQ-30 subscale, Metacognitions Questionnaire 30 Uncontrollability and danger subscale; CCL-A, Cognitions Checklist-Anxiety Subscale; GAD-7, Generalized Anxiety Disorder Scale-7; ªopen-ended questions about participant perceptions of the treatment models

A schedule of enrollment, interventions, and assessments is presented in Fig. 1.

### Sample size {14}

The sample size calculation is based on an effect size estimate for the primary outcome; change in core symptoms of principal diagnosis from baseline to post-treatment. The most recent meta-analysis comparing MCT and CBT reported a pooled between-group effect size of *g* = 0.43 across disorders [22]. However, a previous meta-analysis showed that effect sizes varied greatly across disorders [18]. Notably, only negligible effect sizes were obtained for depression (*g* = 0.04–0.05), while effect sizes for anxiety disorders varied from moderate to large (*g* = 0.62–2.19). In the only study to date comparing tMCT to CBT for anxiety disorders, an effect size of *d* = 0.70 was obtained [23]. Thus, an expected between-group effect size of *d* = 0.70 may be considered a quite conservative estimate. Using the power calculation tool G*Power [68], with 80% power, an estimated between-group effect size of *d* = 0.70 and an alpha value of 0.05 (two-tailed), a total sample size of 68 participants is required. To account for an expected dropout rate of 20% [69], the total sample size is increased to 86 participants (43 per condition). The sample size for the qualitative study is determined using a “pragmatic saturation” criterion [70].

### Recruitment {15}

Participants will be recruited consecutively. Potentially eligible patients will be referred to therapists for assessment of eligibility criteria, principal and any comorbid diagnosis. To facilitate recruitment, participants may be recruited from primary study sites and other care centers within the same clinic.

## Assignment of interventions: allocation

### Sequence generation {16a}

To ensure an even distribution of each principal diagnosis across treatment conditions, participants will be individually stratified by principal diagnosis prior to randomization. A list of random numbers will be generated for each diagnosis and site using the online service www.sealedenvelope.com [71]. The randomization list will be created in blocks of various sizes, and the order of block size is random.

### Concealment mechanism {16b}

Sequentially numbered, opaque, sealed envelopes containing the random numbers will be used to allocate participants to tMCT or CBT. Researchers, therapists, participants and independent assessors will be blinded to the allocation sequence.

### Implementation {16c}

The allocation sequence will be generated by an individual independent of the trial. Therapists will enroll participants and assign interventions by opening the next envelope in the sequence for the participants’ primary diagnosis.

## Assignment of interventions: blinding

### Who will be blinded {17a}

Blinding of participants and therapists to treatment allocation is not feasible. Post-treatment assessors will be blinded to treatment condition and participants will be instructed not to disclose their allocation. To check the integrity of blinding, post-treatment assessors will be instructed to record whether allocated treatment is accidentally revealed and, if not, to guess the treatment allocation. Researchers will be blinded to treatment allocation in the analysis phase at all assessment points. Blinding will be broken after analyses are finalized.

### Procedure for unblinding if needed {17b}

Unblinding procedures are not applicable as participants and therapists will not be blinded.

## Data collection and management

### Plans to promote participant retention and complete follow-up {18b}

To promote retention, participants will be provided with comprehensive information prior to consent regarding the treatments, the randomization procedures and the practical requirements of participation. During treatment, participants will be reminded of each session via text message 2 days prior. If participants do not complete online measures on the day they are administered, e-mail reminders will be sent for 3 days during weekly assessment points and for 5 days for all other assessment points. Participants who discontinue treatment prematurely will be asked if willing to complete the post-treatment assessment and to provide reasons for discontinuation. All instances of discontinuation and protocol deviation will be systematically documented.

### Data management {19}

Data management in this trial will follow the European General Data Protection Regulation [72] as well as guidelines from Karolinska Institutet and the public health care services in Stockholm regarding management of personal data. To ensure data collection is conducted in line with the study protocol, regular on-site monitoring will be conducted by the first author, initially every 2 weeks during the first 2 months at each site and thereafter monthly. The monitoring will follow a structured monitoring checklist.

Data will be collected online, with measures administered via a secure web-based assessment tool. Extracted electronic data will be stored on secure servers. Only the principal investigator and the research team will have access to the final dataset. Range checks for data values will be conducted to optimize data quality. Data will be stored for a minimum of 10 years.

### Confidentiality {27}

To protect confidentiality, all personal data will be pseudonymized and stored under a unique study-ID. A code key linking the study-ID to personal identifiers will be stored separately from the dataset. Access to the code key will be restricted to the first and last author. Data will be presented at the group level in future publications and in the dissemination of results to prevent the identification of individuals. In the qualitative study, quotes from participant interviews will be used. All quotes will be managed in a way that ensures participants remain anonymous and cannot be identified.

### Plans for collection, laboratory evaluation and storage of biological specimens for genetic or molecular analysis in this trial/future use {33}

Not applicable as no biological specimens are collected in this trial.

## Statistical methods

### Statistical methods for primary and secondary outcomes {20a}

Multilevel modelling will be used to estimate between-group effect sizes on primary and secondary outcome measures. A single model including data from all four assessment points will be fitted for each outcome. Fixed effects will include treatment, time, treatment × time, and participant baseline covariates. To account for clustering of patients within therapists, the model will include a random intercept for therapist and a random intercept for patient nested within therapist. Standardized effect sizes for between-group effects will be calculated as Cohen’s *d* for multilevel models with 95% confidence intervals [73] for each contrast of interest (pre- to post-treatment, post-treatment to 6- and 12-month follow-up, respectively). To compare effects across disorders, scores on disorder-specific symptom measures (i.e., the PSWQ, LSAS-SR, and PCL-5) will be standardized by calculating *z*-scores.

Based on *z*-scores of the disorder-specific outcome measures, treatment response will be assessed using the reliable change index [74]. Furthermore, combining reliable change and cut-off values of the PSWQ, LSAS-SR, and PCL-5, respectively, participants’ clinical status at post-treatment will be categorized as recovered, improved, unchanged or deteriorated [74]. Clinical status will also be assessed in terms of remission at post-treatment, defined as no longer meeting diagnostic criteria for the principal diagnosis according to the DIAMOND. Differences in clinical status between conditions will be investigated using chi-square tests.

Mediation analysis will be conducted by disaggregating potential mediators assessed using the MCQ-30 uncontrollability and danger subscale, the CCL-A and the GAD-7 to their within- and between-person components [75]. To establish a temporal sequence between mediator and outcome, mediator scores will be lagged relative to outcome.

In the health economic evaluation, an incremental cost-effectiveness ratio will be calculated as the ratio between the difference in costs relative to the difference in disorder-specific symptom measures and quality-adjusted life years between treatments. In addition, net monetary benefits will be calculated at different willingness-to-pay thresholds. Results are presented using incremental cost-effectiveness ratio planes and cost-effectiveness acceptability curves. The health economic evaluation will be conducted from both a health care perspective (direct costs) and a societal perspective (direct and indirect costs).

The qualitative interviews will be recorded, transcribed verbatim, and analyzed using reflexive thematic analysis [76]. Qualitative data will be analyzed separately from quantitative data.

### Methods for additional analyses (e.g., subgroup analyses) {20b}

Potential moderators of treatment effects will be examined (e.g., demographic variables, baseline symptom levels, etc.) by multilevel modelling, including a three-way interaction term of treatment × time × moderator.

### Methods in analysis to handle protocol non-adherence and any statistical methods to handle missing data {20c}

Data from all randomized participants will be used, following the intention-to-treat principle. In multilevel modeling, missing data do not pose a problem because model parameters are estimated using available data.

### Plans to give access to the full protocol, participant-level data, and statistical code {31c}

There are no plans for public availability of participant-level data or statistical code. Data, statistical analysis, and other materials can be made available upon reasonable request, subject to approval of the participating clinics.

## Oversight and monitoring

### Composition of the coordinating centre and trial steering committee {5d}

The first author will serve as the trial coordinator, overseeing the trial with the principal investigator. Regular meetings will be held with therapists and other staff with daily involvement in the trial.

### Adverse event reporting and harms {22}

No trial-specific anticipated harms are identified that are not shared with other research enterprises on psychological treatment. In principle, all psychological treatments can cause participants to experience a temporary increase in psychological distress initially. However, this typically declines over the course of treatment. Patient status and risk for suicide will be continuously monitored and evaluated during treatment by their assigned therapists. Because the trial will be conducted in a routine care setting, other staff are easily contacted to intervene according to clinical guidelines in cases of acute deterioration, such as an elevated risk of suicide. Therapists, as well as other staff, will be instructed to report any serious adverse events (i.e., suicide, suicide attempt, self-harm, or inpatient admission) immediately upon discovery to the research team. Such events will be documented and reported in future trial publications.

### Composition of the data monitoring committee, its role, and reporting structure {21a}

No data monitoring committee is appointed in this trial as it does not involve any trial-specific risk of harm beyond those common in research on psychological treatment. Oversight responsibilities will be assigned to the principal investigator and the trial coordinator.

### Interim analyses {21b}

No interim analyses are planned, and it is not deemed necessary to arrange stopping guidelines to terminate the trial prematurely. Participants in active treatment will be continuously monitored by their assigned therapists.

### Frequency and plans for auditing trial conduct {23}

There are no planned independent trial audits.

### Plans for communicating important protocol amendments to relevant parties (e.g., trial participants, ethical committees) {25}

Any substantial modifications to the study protocol will require additional application to the Swedish Ethical Review Authority and will be communicated to participants, therapists and other staff, as well as reported through updates to the trial registration and in future publications.

### Dissemination plans {31a}

Results will be disseminated via open-access, peer-reviewed publications and presentations at national and international conferences. Reporting will follow appropriate guidelines, such as the CONSORT-NPT [77] for the randomized controlled trial and the COREQ [78] for the qualitative study. Participants will be informed to contact the principal investigator if they wish to access trial results.

## Discussion

Anxiety disorders are highly prevalent globally [1] and associated with significant personal and societal consequences [3–6]. MCT has received support as an effective treatment for anxiety disorders, with results indicating that MCT in both a disorder-specific and transdiagnostic format may be superior to CBT [22, 23]. However, with only one previous adequately powered study comparing tMCT and CBT for anxiety disorders, further studies are warranted. This trial aims to evaluate the short- and long-term effects of tMCT versus disorder-specific CBT for anxiety disorders, as well as to compare the cost-effectiveness of the treatments. Additionally, the trial will examine metacognitions and cognitions as possible mediators of treatment response to contribute to the knowledge base on mechanisms of change in both MCT and CBT. Also, this trial will provide highly needed qualitative data that could facilitate implementation of MCT in routine care and advise future treatment development.

Despite extensive planning and preparation, there are some limitations to this trial. Firstly, it is not possible for therapists or participants to be blinded to treatment condition during the treatment phase. However, assessors will be blinded during the post-treatment assessment interview and researchers are blinded during analysis to reduce the risk of bias in these steps. Secondly, outcomes rely primarily on self-report measures, and potential reporting bias cannot be ruled out. However, this is expected to affect both treatment groups equally. Clinician-administered instruments will be used for some outcomes, providing complementary information but not directly addressing this limitation. Thirdly, there might be a risk for therapist effect bias with therapists providing either tMCT or CBT instead of both treatments. However, providing either treatment can also have benefits, such as minimizing the risk of treatment allegiance effects [79]. If therapists provide both treatments, there is a risk that therapists’ potential favorable attitudes towards one treatment could influence results. In addition, although tMCT and CBT use some similar treatment techniques, they rely on different theoretical models with different implications for treatment delivery. Switching between treatments while maintaining treatment integrity could therefore be challenging.

To the best of our knowledge, this trial involves only the second study on tMCT compared to disorder-specific CBT for anxiety disorders and the first to be conducted in an outpatient psychiatric care setting. The results of this trial will provide valuable information on the effects of MCT in a transdiagnostic format and contribute to improving the knowledge of psychological treatment for anxiety disorders. This trial can also provide valuable insights into what makes psychological treatments work, how to facilitate treatment implementation, and guide future treatment development and health economic-based decision-making in the health care system.

### Trial status

Protocol version 1, September 29th, 2026. Recruitment started August 27th, 2025, and completion is anticipated by December 31 st, 2028.

## Data Availability

Results from the studies included in this trial will be communicated in future publications. Only the principal investigator and research team will have access to the final trial dataset. Data, statistical analysis and other materials can be made available upon reasonable request, subject to approval of the participating clinic.

## Abbreviations

CAS: Cognitive attentional syndrome
CBT: Cognitive behavioral therapy
CCL-A: Cognitions Checklist-Anxiety Subscale
CSR: Clinical Severity Rating
DIAMOND: Diagnostic Interview for Anxiety, Mood, and OCD and Related Neuropsychiatric Disorders
DSM-5: Diagnostic and Statistical Manual of Mental Disorders, fifth ed.
EQ-5D-5L: EuroQol Five Dimensions Five Levels
GAD: Generalized anxiety disorder
GAD-7: Generalized Anxiety Disorder 7-item scale
LSAS-SR: Liebowitz Social Anxiety Scale-Self-Report
MCQ-30: Metacognitions Questionnaire-30
MCT: Metacognitive therapy
PCL-5: Posttraumatic stress disorder Checklist-5
PHQ-9: Patient Health Questionnaire-9
PSWQ: Penn State Worry Questionnaire
PTSD: Posttraumatic stress disorder
S-REF: Self-regulatory executive function model
SAD: Social anxiety disorder
SWLS: Satisfaction With Life Scale
TIC-P: Treatment Inventory of Costs in Patients with psychiatric disorders
tMCT: Transdiagnostic metacognitive therapy
WHODAS: World Health Organization Disability Assessment Schedule
